# Thermophysical Molding Treatments on Thick Wood Veneer

**DOI:** 10.3390/polym14173516

**Published:** 2022-08-27

**Authors:** Yaohui Ji, Yue Qi, Rongxian Zhu, Hongxia Ma, Yahui Zhang, Wenji Yu

**Affiliations:** 1 Research Institute of Wood Industry, Chinese Academy of Forestry, Xiang Shan Road, Haidian District, Beijing 100091, China; 2 Guangdong Academy of Forestry, 233 Guangshan 1st Road, Tianhe District, Guangzhou 510520, China

**Keywords:** thermophysical molding treatment, wood, thick veneer, surface, properties, wettability, roughness, chemical change

## Abstract

Thermophysical molding (TPM) treatments can significantly improve the surface properties of thick wood veneer. To understand the effects of TPM treatments on the surface properties of thick veneer, the roughness, contact angles, and chemical changes were determined. The results indicated that the roughness of the thick veneer decreased when the temperature and the duration increased. The contact angles decreased when the temperature increased, resulting in better wettability. X-ray photoelectron spectroscopic (XPS) results provided information about the significant chemical changes in the surface with different TPM temperatures of 160–190 °C and durations of 5–11 min. Increases in temperature and duration increased the C content and decreased the O content during the treatment process. The most significant changes in the thick veneer that resulted from increasing the temperature and the duration were the increase in the C1 component and the decrease in the C2 component. Thus, the oxygen to carbon (O/C) ratio decreased and the ratio of aromatic carbon to aliphatic carbon (C1/C2) notably increased with the increasing TPM temperature. The TPM duration slightly affected the O/C ratio, but it had a stronger linear relation with the C1/C2 ratio. Additionally, the C1/C2 ratio and the O/C ratio had a linear statistical relationship with the initial wettability. These findings could provide useful information for the future utilization of thick veneers treated with TPM.

## 1. Introduction

Plywood is the most productive species of wood-based panel products, and it is greatly utilized in furniture and building processes [1,2,3]. Traditional plywood is composed of rotary veneer, which is laid up crosswise to form the desired thickness in the range of 1.7 mm to 4.0 mm. Increasing the thickness of the veneer, which can not only reduce the use of adhesives, but also enhance the visual effect of solid wood, is one of the directions of wood veneer utilization [4]. Therefore, the development of wood-based composite products made from thick veneers with high added value has become a new research topic in the wood industry.

The enhancement of veneer units is one of the most important ways to upgrade traditional composite wood materials. Internal stress is related to the thickness of the veneer, and an increased thickness could increase stress and deformation, resulting in poor stability of a product’s quality. The fact that raw wood veneer can be pressed under the action of mechanical and thermal loads has been known for a very long time and has been commonly used in industrial products [5]. The reason for treating the veneer is to improve some of its intrinsic properties, such as dimensional stability, resistance to micro-organisms, stress relaxation during the formation of wood-based composites, veneer production, and wood fracture resistance. Moreover, this was the first thermal-hydro-mechanical (THM) treatment that had an important advantage over the chemical treatments since the products of the THM veneer treatment are particularly environmentally friendly [6,7]. This process can also release internal stress and soften the veneer so that it can be easily glued with resin. Advanced investigations of the THM treatment have been reported and showed that the THM treatment of thin wood specimens at 200 °C for only 4 min was sufficient to almost totally eliminate the shape memory of densified wood [8,9,10]; therefore, the THM treatment of wood was limited to 300 °C, due to the severe degradation of the strength of the material [11,12,13]. However, the problems related to wood veneer, such as the development of cracks, deformation, and energy consumption, have yet to be completely solved. THM treatments usually have a long processing duration and require an additional steam injection [14,15,16], which results in a great energy loss, a high demand for the condition of the equipment, a difficult processing procedure, and it also causes degradation of the chemical composition of the wood, which, in turn, reduces the mechanical properties of the material itself. Bekhta and Krystofiak (2016) tried to shorten the treatment duration and investigated the effects of short-term THM treatments on the surface properties of wood veneers; however, the study was limited to thin veneers that were 1.5 mm thick [17]. Additionally, as glue or paint is applied to the wood surface in the manufacturing of wood composites, the surface properties of wood and wood veneers are vital, such as roughness, wettability, chemical composition, etc. [17,18]. Thus, some scholars have done research on the surface properties of THM veneers, but their studies were all focused on thin veneers and did not involve thick veneers.

However, thick veneers are thicker and have problems such as high surface roughness, more cracks due to rotary cutting, and poorer dimensional stability, which all affect subsequent processing (gluing, coating, etc.) [19,20,21]. The traditional THM method, which uses an external injection of hot steam to soften the inside of the thick veneer, is less efficient. Thus, the THM treatment needs to be improved for thick veneers so it is comparable to the first treatments, and so that its main purpose is not only to release the internal stress but also to keep the veneer surface constantly flattened for the elimination of the compression-set-recovery effects or shape memory.

In this study, a THM treatment was developed into the thermophysical molding (TPM) technique, which combined a thermal treatment, physical action, and a molding technique, to achieve stress relief and even an accurate control of the surface properties of thick veneer. In addition, the water inside the high-moisture-content thick veneer itself was used as the source of steam in the TPM method, which not only made the veneer dry quickly but also achieved the effect of softening the veneer to reduce its internal stress. This reduced the production processes and greatly improved the processing efficiency of the thick veneer; thus, the effects of the TPM processing parameters, such as temperature and duration, on the various properties needed to be investigated. The appropriate parameters of the TPM treatment were discussed in this research, and the surface properties of thick veneer including roughness, wetting ability, contact angle, and surface free energy were then investigated by changing different parameters during the TPM treatment; these could be vital for subsequent gluing techniques and manufacturing processes of thick veneer board. The chemical properties of the veneer surface under X-ray photoelectron spectroscopic (XPS) measurement were examined and evaluated.

## 2. Materials and Methods

### 2.1. Materials

Poplar (*Populus* L.), which was seven years old, with a diameter of 30 cm, was obtained from the industry in Shandong province. In this study, thick veneers, with thicknesses of 8 mm and taken from a sapwood, were rotary cut from fast-growing poplar wood. The average moisture content of the obtained thick veneers was 26.42%. Formamide (methanamide) and ethylene glycol (ethane-1,2-diol), purchased from Sigma-Aldrich (Sigma-Aldrich (Shanghai) Trading Co., Ltd., Shanghai, China), were used as received.

### 2.2. Evaluation of the Properties of Thick TPM-Treated Veneer

**Figure 1:**
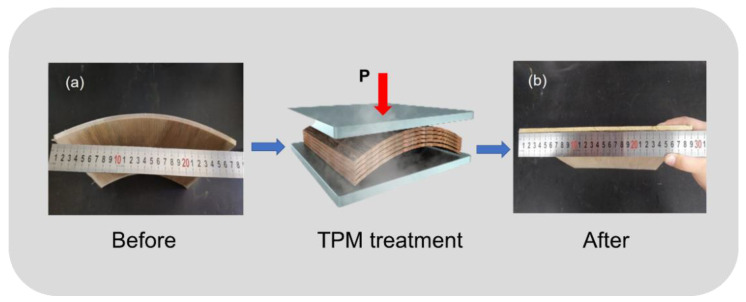
Thermophysical molding treatment on thick veneer: (**a**) untreated sample and (**b**) treated sample.

Thick veneer was treated with a constant pressure of 0.33 MPa during the TPM process (Figure 1). The different temperatures and durations were compared for the evaluation of the surface properties of the thick TPM-treated veneers. When the temperature was set to 180 °C, we selected four duration conditions of 5, 8, 11, and 14 min to prepare the TPM-treated veneers. When the processing duration was 8 min, the performances of the TPM-treated veneers under the four temperature conditions (160, 170, 180, and 190 °C) were discussed. Therefore, seven types of processing for preparing the TPM-treated veneers were performed. All specimens were conditioned in a chamber at 20 ± 2 °C with a relative humidity (RH) of 65 ± 5% for 2 weeks before being tested. The above experimental specimens were used for testing roughness, contact angle, wetting ability, and XPS studies.

#### 2.2.1. Roughness

The thick veneers were discussed in order to assess their roughnesses according to the standard GB/T 1031-2009. The surface roughness meter (TIME 3230, Beijing Time Ricon Technology Co., Ltd., Beijing, China) was applied to six spots on the veneer surface for testing. The measurements of Ra and Rz were repeated at least five times on the different specimens. Considering the inaccuracy of the roughness measurements due to the large cracks on the loose side, we chose the tight side of the veneer for the roughness measurements and their comparison.

#### 2.2.2. Contact Angle Measurement

Thick TPM-treated veneer samples (50 by 50 mm square) were used for the measurements performed by a contact angle analyzer (JC2000D, Shanghai Zhongchen Digital Technology Equipment Co., Ltd., Shanghai, China). The measurement of a contact angle was carried out by the sessile drop profile method. The contact angles between each droplet and the specimen surface were measured both on the left side and the right side of the droplet, and the mean contact angles were automatically calculated. Water and phenol-formaldehyde (PF) resin were mainly discussed in this study.

#### 2.2.3. Wettability

The specimens for the wettability test were prepared the same as in Section 2.2.2. The surface-free-energy parameters of three liquids are referred to in the literature [22]. Formamide (methanamide) and ethylene glycol (ethane-1,2-diol) were used to study the acidic and basic characteristics of the thick TPM-treated veneer surfaces, respectively. The contact angle data of distilled water in Section 2.2.2 could be directly used as the calculation for this part.

The solid–liquid surface free energy can be expressed using the Young equation.

(1) $$\gamma_{L}\cos\theta =\gamma_{S}+\gamma_{\mathrm{SL}}$$

where γ_L_ is the solid–liquid surface free energy, γ_S_ is the solid surface free energy, and γ_SL_ is the interface energy.

According to Van Oss’s theory [23], the surface free energy is calculated using the Lifshitz–van der Waals equation as below:

(2) $$\gamma =\gamma_{\mathrm{LW}}+\gamma_{\mathrm{AB}}$$

where γ is the surface free energy, γ_LW_ is the Lifshitz–van der Waals-based surface free energy, and γ_AB_ is the acid–base-based surface free energy.

(3) $$\gamma_{\mathrm{AB}}=2\sqrt{\gamma_{A}\gamma_{B}}$$

where γ_A_ is the acid-based surface free energy and γ_B_ is the base-based surface free energy.

### 2.3. XPS Analysis

Samples of size 5 mm × 5 mm × 1 mm (longitudinal × width × thickness) were prepared. XPS measurements were performed on an XPS spectrometer (Kratos Axis Ultra, Kratos Analytical, Manchester, UK). Spectral fitting and component analysis were performed using high-resolution spectra, and the number of components and their binding energy positions were obtained from previous studies [24,25]. The XPS data were collected using a K-alpha XPS system with an aluminum Kα X-ray source. The base pressure in the analytical chamber was less than 5 × 10^−7^ Pa, and the survey scans’ spanning binding energies from 1200 to 0 eV were collected using a constant pass energy of 30 eV and a step interval of 0.050 eV. 

### 2.4. Statistical Analysis

To detect the difference in roughness among the samples prepared using different temperatures and durations, analysis of variance (ANOVA) was performed using SPSS (IBM SPSS software version 24, SPSS Inc., Chicago, IL, USA)—*p* being the significance of the differences, while defining 5% as the significance level (*p* < 0.05).

## 3. Results and Discussion

### 3.1. Roughness of TPM-Treated Veneers

**Figure 2:**
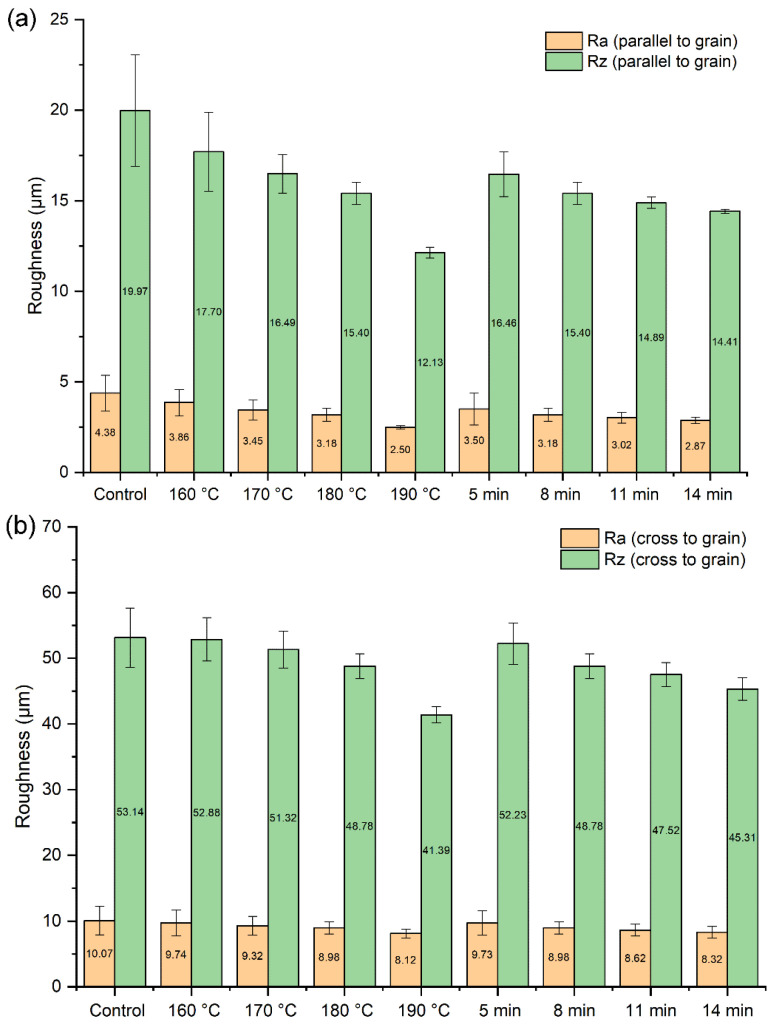
Effect of TPM temperature and duration on the roughness parallel to grain (**a**) and the roughness on the cross grain (**b**) of the thick veneer surfaces.

Figure 2 show the influence of the TPM temperature and the duration on the roughness of the veneers. The roughness values (Ra and Rz) parallel to the grain were significantly lower than those on the cross grain. The highest roughness parallel to the grain, 4.38 (Ra) and 19.97 (Rz) μm, and the highest roughness on the cross grain, 10.07 (Ra) and 53.14 (Rz) μm, were detected in the control specimens. As for different TPM temperatures (consistent duration: 8 min), the lowest surface roughness in the TPM-treated veneers were 2.50 (Ra) and 12.13 (Rz) μm parallel to the grain and 8.12 (Ra) and 41.39 (Rz) μm on the cross grain during the TPM treatment at 190 °C, which showed an obvious increase in smoothness compared with the control experiment. With the increase in temperature, the roughness decreased in the tested specimens. Compared with flattened TPM-treated veneers at the different TPM treatment durations (consistent temperature: 180 °C), the treated specimens at 14 min showed the lowest Ra and Rz values and had the smallest standard deviation. For the roughness parallel to the grain, the Ra and Rz values of the samples prepared at 5 min were 1.22 and 1.14 times higher than those at 14 min, respectively, while for the roughness on the cross grain they were 1.17 and 1.15 times higher, respectively. Based on the results of increasing temperature and duration, the roughness values showed a generally declining trend. In addition, only the temperature showed clear effects on the Rz values parallel to grain (*p* = 0.011 < 0.05) based on the multivariate ANOVA results.

Whether cross or parallel to the grain, the Ra and Rz values of the thick veneers after the TPM treatment were lower than those of the untreated specimens, suggesting a decrease in roughness. These results were in agreement with some previous studies [26]. They reported that the roughness of common alder and wych elm wood decreased with increasing treatment temperature and treatment duration. Another study examined the surface roughness of redbud maple and demonstrated that the average roughness of the samples decreased by up to 15% with increasing heat treatment duration [27]. Overall, the decrease in roughness is very important for many applications of solid wood, including thick wood veneers. An increase in temperature and duration could produce high-quality thick veneer surfaces.

### 3.2. Influence of TPM Parameters on the Wetting Performance of the Veneer Surfaces

**Figure 3:**
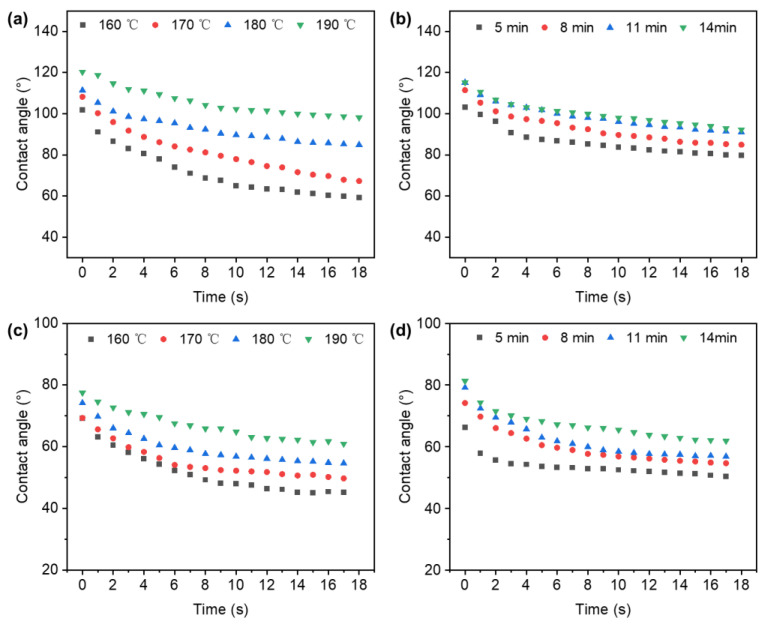
(**a**) Contact angles tested by water in the TPM-treated veneers at different temperatures; (**b**) contact angles tested by water in the TPM-treated veneers at different durations; (**c**) contact angles tested by PF resin in the TPM-treated veneers at different temperatures; and (**d**) contact angles tested by PF resin in the TPM-treated veneers at different durations.

Wettability is important for adhesion in wood bonding; the surface is wetted and then the cellular structure is penetrated to establish an initial contact between the molecules of the adhesive and the cell wall components of wood [28]. In this study, water and phenol formaldehyde (PF) resin were tested to determine the contact angle in the veneer surfaces that were treated with different TPM temperatures and durations. In Figure 3, the contact angles of both the water and the PF resin showed a positive correlation with the TPM temperature and duration. As the temperature increased, the surface of the thick TPM-treated veneer became smoother with the larger contact angle, due to the duration increase. Compared with TPM duration, the temperature showed a greater influence on the contact angle. The above results could be attributable to the degradation of hemicelluloses on the veneer surface during the TPM process, which results in a reduction in OH bonds and the O-acetyl group, and the subsequent cross-link formation between the wood fibers makes the wood more hydrophobic [29]. Furthermore, the wetting characteristics of wooden surfaces are highly influenced by the surface roughness, which is determined by Wenzel’s theory. It is known that surface roughness has a magnified effect on surface wettability. Namely, roughness has an enhanced effect on the veneer surface, which shows that an increase in roughness could lead to an increase in the hydrophilicity of the hydrophilic surface or hydrophobicity of the hydrophobic surface. 

Table 1 shows the related parameters of the surface properties. Surface free energy (γ) and acid–base-based surface free energy (γ_AB_) notably decreased from 45.54 to 5.60 mJ/m^2^ and 15.52 to −20.19 mJ/m^2^ during temperatures from 160 to 190 °C, respectively, indicating an obvious effect of temperature on surface properties. Regarding the increase in duration from 5 to 14 min, the apparent variation of γ and γ_AB_ was observed to decrease from 25.62 to 1.61 mJ/m^2^ and −1.35 to 20.38 mJ/m^2^, respectively. These results revealed that the highest temperature and longest duration resulted in the lowest surface free energy and surface polarity. The value of γ_LW_ was calculated by γ and γ_AB_ and gradually decreased from 30.20 to 25.78 mJ/m^2^ and 26.98 to 21.99 mJ/m^2^ along with increasing temperature and duration, respectively.

Higher surface free energy could lead to better wettability due to the variation of contact angles, which was determined by chemical functional groups during the process of increasing temperature and duration [30]. Dihydroxylation happened on the surface of the wood veneers resulting in a reduction in water absorption at the higher temperature treatments [31]. Accordingly, these results suggested that appropriate temperatures and durations were important for the improvement of wettability, and made the veneer surfaces easier to spread adhesives onto and for the adhesives to penetrate the wood during the manufacturing process.

### 3.3. Chemical Components on the Surfaces of the TPM-Treated Veneers

**Figure 4:**
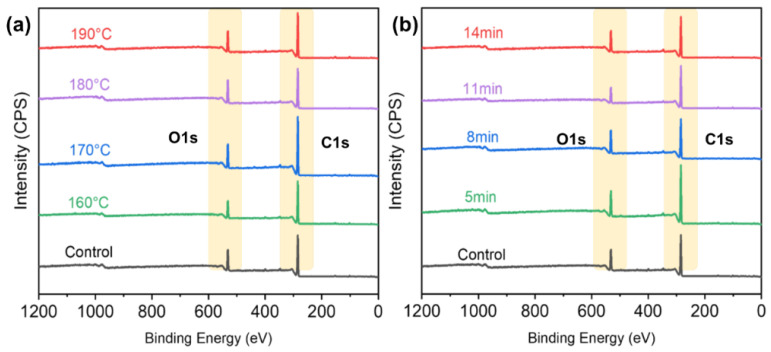
XPS survey spectra of different TPM temperatures (**a**) and durations (**b**).

XPS analysis has been demonstrated as a basic method for characterizing chemical components of material surfaces concerning interfacial phenomena [24]. XPS survey spectra of different TPM temperatures and durations are shown in Figure 4, respectively. As expected, specimens with different temperatures and durations consisted mainly of electrons from carbon (C1s), electrons from oxygen (O1s) atoms, and the associated Auger electron peaks. Comparing survey spectra of samples with different temperatures and durations, the carbon C1s peak increased and the oxygen O1s peak decreased due to composition degradation in the thick veneer surfaces [32,33]. Moreover, the oxygen to carbon ratio (O/C ratio) and the aromatic carbon to aliphatic carbon (C1/C2) ratio were also calculated.

**Figure 5:**
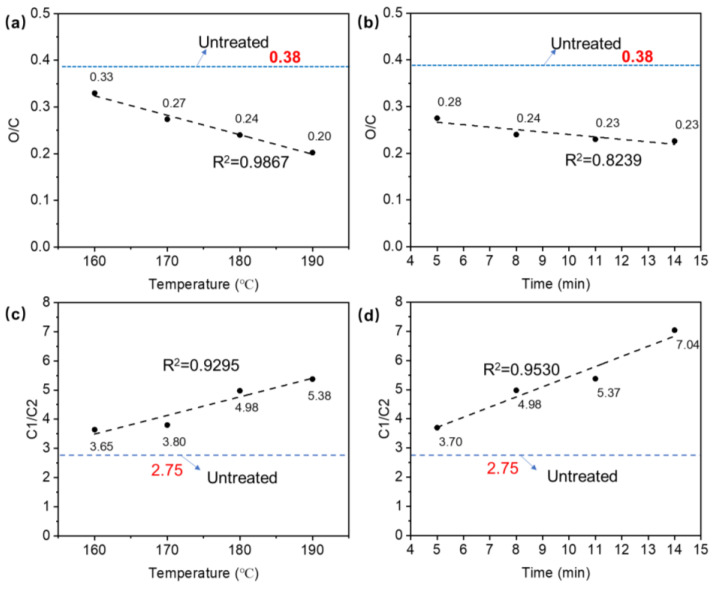
Effect of temperature and duration on the O/C ratio and the C1/C2 ratio of TPM-treated thick veneer: (**a**) correlation between the temperature and the O/C ratio; (**b**) correlation between the duration and the O/C ratio; (**c**) correlation between the temperature and the C1/C2 ratio; and (**d**) correlation between the duration and the C1/C2 ratio. The dashed line in each figure represents the ratios of the untreated samples.

The degradation of cellulosic materials and polymers can be detected by a change in the O/C atomic ratio [24]. Using the total areas of peaks of different components, the O/C ratio can be quantitatively determined. Figure 5 shows that the O/C ratio decreased from 0.33 to 0.20 after the temperature was increased from 160 to 190 °C during the THM treatment. The decreased O/C ratio was attributed to the important degradations occurring during the TPM treatment. This was in agreement with the results of a previous study [28], which showed that high temperatures ranging from 160 to 180 °C decreased the O/C ratio of wood during treatment. It was also reported that the O/C ratio decreased from 0.55 to 0.44 after the high-temperature treatment of beach wood, and this decrease in O/C ratio appeared to be closely related to carbohydrate (cellulose and hemicellulose) degradation, leading to the formation of volatile by-products with a lower oxygen content that resulted from the dehydration of polymers that were initially present in the wood [24]. Chu et al. [34] also revealed that higher temperatures led to a reduction in the O/C ratio, which could further improve the degradation of hemicellulose and the regeneration of lignin. The O/C ratios of all the TPM-treated samples were lower than that of the untreated samples’ surfaces (0.38). Furthermore, the O/C ratio slightly decreased from 0.28 to 0.23 when the TPM-treatment duration was increased from 5 to 14 min (Figure 5b). The O/C ratio slightly decreased as the THM duration changed from 8 min, during which it remained constant even when the duration was increased to 11 and 14 min. Overall, it was revealed that the THM duration extension did not affect the O/C ratio significantly.

**Figure 6:**
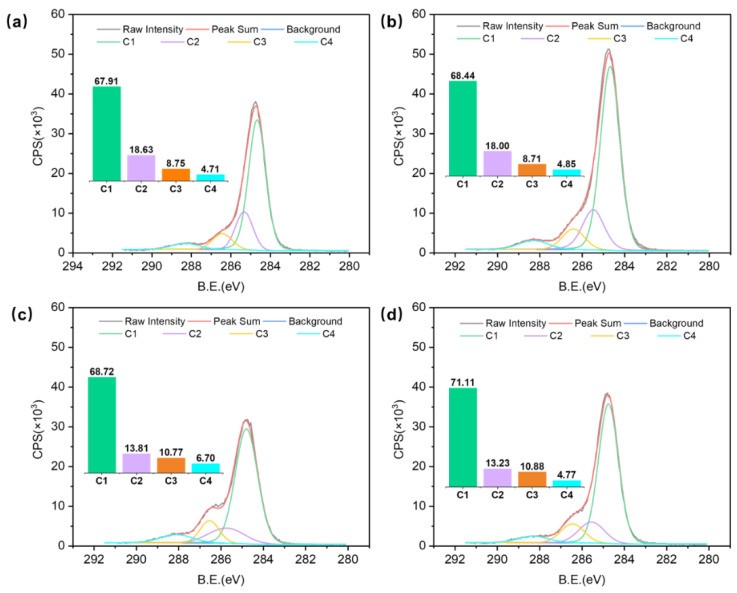
Effect of TPM temperature on the C1s’ peak components in the following samples: (**a**) 160 °C; (**b**) 170 °C; (**c**) 180 °C; and (**d**) 190 °C. The bars with different colors inside of the figure represent the values of C1, C2, C3, and C4, respectively.

**Figure 7:**
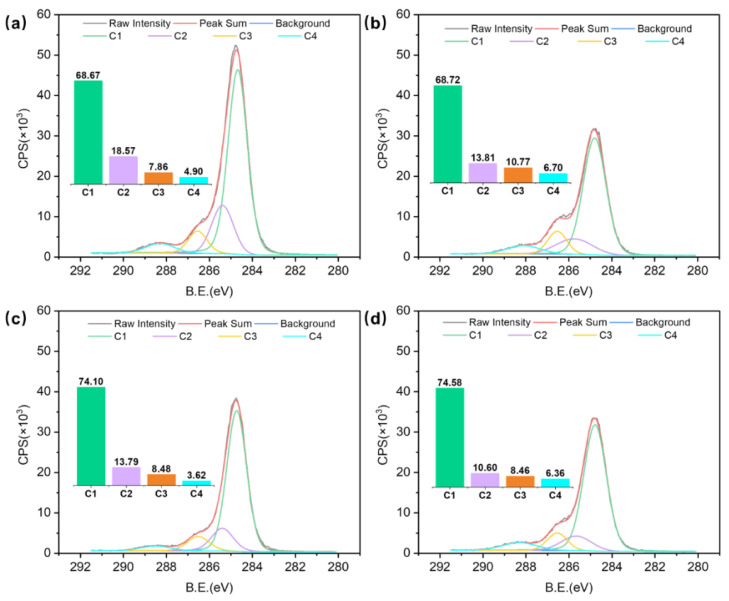
Effect of TPM duration on C1s’ peak components in the following samples: (**a**) 5 min; (**b**) 8 min; (**c**) 11 min; and (**d**) 14 min. The bars with different colors inside of the figure represent the values of C1, C2, C3, and C4, respectively.

High-resolution scans of C1s were decomposed into four components for the experimental specimens: C1 (graphite (C-C) at 284.7 eV), C2 (C-OH at 285.6 eV), C3 (C=O at 286.5 eV), and C4 (COOH at 288.3 eV), respectively. Differences in peak areas, which indicated the surface changes, are present in Figure 6 and Figure 7. The most significant changes in the thick veneer that resulted from increasing the temperature and the duration were the increase in the C1 component and the decrease in the C2 component. The contributions of the C1 and C2 peaks were more important than the C3 and C4 peaks, indicating that they had higher concentrations at the surface of the samples. As the TPM temperature increased, the peak area proportion of C1 increased from 67.91% to 71.11%, while that of C2 decreased from 18.63% to 13.23%. In addition, C1 increased from 68.67% to 74.58%, while C2 decreased from 18.57% to 10.60%, with increasing TPM duration, respectively. The results revealed that a notable change appeared at the TPM temperature of 180 °C (Figure 6). The samples treated with a constant temperature of 180 °C showed sensitive variations when prolonging the TPM duration, which had a significant effect on their chemical compositions. The C1 component is associated with the presence of lignin on the veneer surface, and the C2 component mainly originates from cellulose and hemicellulose [31]. Thus, the TPM treatment could be partially attributed to an increase in lignin content due to the preferential degradation of hemicellulose, leading to the formation of volatile by-products, such as furfural or acetic acid [35,36]. The C1/C2 ratio is related to the lignin content of a material based on the presence of aromatic carbon in the lignin [37], which increased by 1.73 and 3.34 with increasing temperatures and durations, respectively (Figure 5c,d). A significant rise was obtained at the TPM temperature of 180 °C and a duration of 14 min. A comparison of the C1/C2 ratios between the thick TPM-treated veneer and the untreated samples showed that all the ratios of the TPM samples were significantly higher than the untreated samples. Furthermore, enhancements of temperature and duration were conducive to changes in the chemical composition.

**Figure 8:**
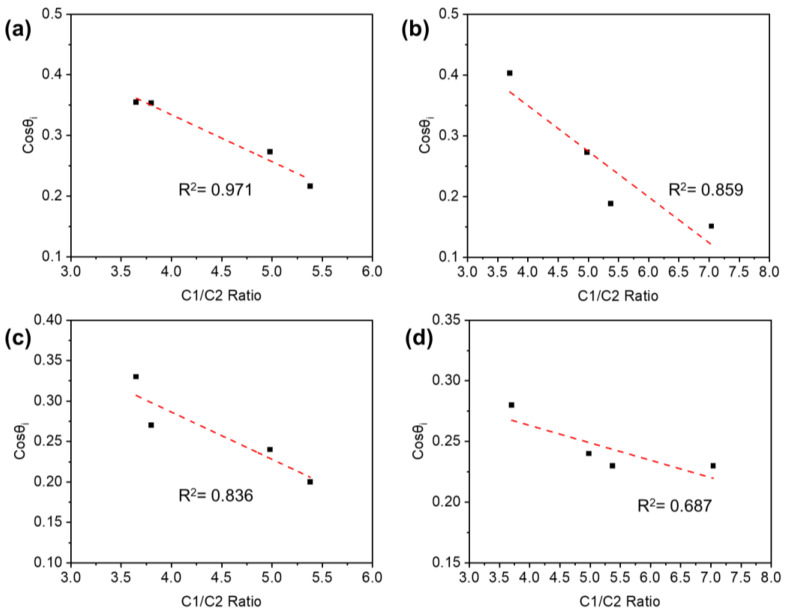
Relationship between initial wettability tested by water and the C1/C2 ratio of the TPM-treated veneer with different temperatures (**a**) and durations (**b**), and tested by PF resin and the C1/C2 ratio of the TPM-treated veneer with different temperatures (**c**) and durations (**d**).

**Figure 9:**
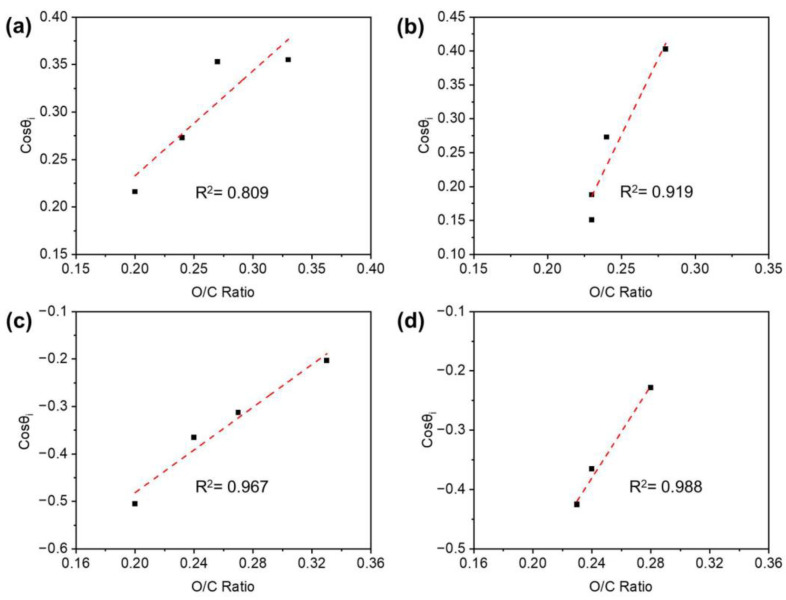
Relationship between initial wettability tested by water and the O/C ratio of the TPM-treated veneer with different temperatures (**a**) and durations (**b**), and tested by PF resin and the O/C ratio of the TPM-treated veneer with different temperatures (**c**) and durations (**d**).

An initial wettability (cos θi) was plotted against the C1/C2 ratio and the O/C ratio to find a possible relationship between the wetting capacity of the TPM-treated veneer surface and the change in chemical composition. In a previous study [28], the cosine function was selected due to the relationship among the interfacial surface tensions of the vapor, moisture, and solid phases, and cos θ is often used as a direct measure of surface wettability. As shown in Figure 8, the C1/C2 ratio had a strong relationship with the initial wettability, which was tested by water. The wettability of the TPM-treated veneer surface decreased when the C1/C2 ratio increased (Figure 8a). Meanwhile, the O/C ratio had a positive correlation with the initial wettability of the veneer surface penetrated by PF resin (Figure 9c,d). Therefore, a linear statistical model explained most of the variability between the initial contact angle and the C1/C2 or O/C ratio.

## 4. Conclusions

The comparative analysis on the surface of the poplar thick veneer revealed an obvious relationship between the parameters of TPM treatment and the surface properties, including roughness, wettability, and chemical composition. The surface roughness was reduced with an increase in temperature and duration, regardless of whether it was cross or parallel to the grain. Increasing the temperature and duration could lead to higher surface free energy, resulting in better wettability. The highest contact angles were found in the higher temperatures and longer durations. Analyzing the changes in chemical composition, the results suggested that the O/C ratio decreased, and the C1/C2 increased with an increase in the TPM temperature and duration. Furthermore, initial wettability had a statistical correlation with chemical composition under a range of TPM temperatures and durations. Comprehensively, the appropriate temperature and duration of a TPM treatment could improve and activate the veneer surface. The results obtained in this study could be used as initial information for adhesive/coating application processes in thick TPM-treated veneers during the preparation of wood-based composites.

## Figures and Tables

**Table 1:** Surface free energy of TPM-treated veneers at different temperatures and duration.

Parameters	Temperature (°C)				Duration (min)			
	160	170	180	190	5	8	11	14
γ (mJ/m^2^)	45.54	33.61	20.74	5.60	25.62	20.74	10.42	1.61
γ_LW_ (mJ/m^2^)	30.02	29.14	26.44	25.78	26.98	26.44	24.97	21.99
γ_AB_ (mJ/m^2^)	15.52	4.47	−5.70	−20.19	−1.35	−5.70	−14.55	−20.38

## Data Availability

The data presented in this study are available on request from the corresponding author.
